# A review on magnesium alloys for biomedical applications

**DOI:** 10.3389/fbioe.2022.953344

**Published:** 2022-08-16

**Authors:** Ting Zhang, Wen Wang, Jia Liu, Liqiang Wang, Yujin Tang, Kuaishe Wang

**Affiliations:** ^1^ School of Metallurgical Engineering, Xi’an University of Architecture and Technology, Xi’an, China; ^2^ Department of Orthopaedics, Affiliated Hospital of Youjiang Medical University for Nationalities, Baise, Guangxi, China; ^3^ State Key Laboratory of Metal Matrix Composites, School of Material Science and Engineering, Shanghai Jiao Tong University, Shanghai, China

**Keywords:** magnesium alloy, biomedical applications, corrosion resistance, surface modification, microsturcture and properties

## Abstract

Magnesium (Mg) and Mg alloys are considered as potential candidates for biomedical applications because of their high specific strength, low density, and elastic modulus, degradability, good biocompatibility and biomechanical compatibility. However, the rapid corrosion rate of Mg alloys results in premature loss of mechanical integrity, limiting their clinical application in load-bearing parts. Besides, the low strength of Mg alloys restricts their further application. Thus, it is essential to understand the characteristics and influencing factors of mechanical and corrosion behavior, as well as the methods to improve the mechanical performances and corrosion resistance of Mg alloys. This paper reviews the recent progress in elucidating the corrosion mechanism, optimizing the composition, and microstructure, enhancing the mechanical performances, and controlling the degradation rate of Mg alloys. In particular, the research progress of surface modification technology of Mg alloys is emphasized. Finally, the development direction of biomedical Mg alloys in the future is prospected.

## 1 Introduction

Biomaterials are used for diagnosing, treating, repairing or replacing damaged tissues, and further enhancing the function of organisms (Liu et al., 2021a). Bone implant materials are an important part of biomedical materials, and about 70%–80% of implants are made of biomedical alloys (Hafeez et al., 2019; Liu et al., 2020c). The demand for biomedical alloys is rapidly increasing as the world population is getting older. The most representative biomedical alloys include stainless steel (Du et al., 2022), cobalt (Co)-chromium (Cr) alloys (Yamanaka et al., 2016; Zhou et al., 2020b), titanium (Ti) and its alloys (Stráskỳ et al., 2022; Zhang et al., 2022), showing great biological and mechanical performances. However, a second surgery is required to remove the aforementioned implant materials after the bone has healed, which is extremely painful to the patient.

Magnesium (Mg) with biodegradability has gradually attracted extensive attention in biomedical filed (Zhang et al., 2021a; Dong et al., 2022a). It not only exhibits mechanical performances similar to human bone (density of 1.74 g/cm^3^ and elastic modulus of 41–45 GPa), but also shows good biocompatibility (Chen et al., 2018; Dong et al., 2022a). Mg is an essential nutrient for the human body to keep healthy, which can promote bone growth, enhances cell adhesion to biomaterials, and assists the differentiation and biomineralization of osteoblasts (Schwalfenberg and Genuis, 2017). Furthermore, Mg alloys are easily corroded in physiological environment due to their active chemical performances, so they can degrade to produce magnesium hydroxide [Mg(OH)_2_] and hydrogen (H_2_) (Johnson et al., 2012; Agarwal et al., 2016). Compared with the other metal implants, the corrosion products of Mg alloys have been proved to be non-toxic and can be excreted through human metabolism (Wang et al., 2020a).

However, the development of Mg alloy for biomedical applications faces great challenges. The high corrosion rate of Mg in the human body leads to extremely rapid degradation, loss of mechanical integrity, and implant failure prior to the healing process (Banerjee et al., 2019). In addition, corrosion products such as H_2_ gas and OH^−^ ions can affect the biocompatibility of material. H_2_ will accumulate in the neighboring tissue in the form of air holes, causing tissue layers to separate (Jung et al., 2019). OH^−^ ions will lead to surface alkalization and potentially damage cells (Chin et al., 2020). Furthermore, the mechanical performances of Mg alloys, including the hardness, ductility, strength, wear resistance, and toughness, should be improved to satisfy various biomedical applications.

Recently, numerous efforts have been made to improve the mechanical and biological performances of Mg alloys, which are mainly divided into metallurgical modification and surface modification. Metallurgical modification is an effective method to optimize composition and microstructure by alloying, composite fabrication, and heat treatment. While it significantly enhances the mechanical performances and degradation resistance, the release of some toxic elements during the process damages neighboring tissues (Li et al., 2021). In comparison, surface modification is a more promising approach of tuning the microstructure and improving performances, which is accomplished by preparing protective coatings or changing the surface features of materials. The mechanical integrity, biodegradability, and biocompatibility of Mg alloys have been improved considerably through various surface modification processes (Lin et al., 2019; Yao et al., 2020; Zhang et al., 2021b). At present, surface modification methods mainly include mechanical, physical, chemical, and biochemical methods (Zhang et al., 2020b). To fully understand the biological and mechanical behaviors of biomedical alloys, it is essential to carried out a thorough analysis of their structures and modification mechanisms.

This review first introduces the development and characteristics of Mg and Mg alloys for biomedical applications, and then compares main methods and technologies for controlling the degradation rate as well as improving the corrosion resistance and biocompatibility of Mg alloys. Furthermore, the performances and applications of biomedical Mg alloys modified by these methods are discussed. Finally, the directions for future research and possibilities are also illustrated.

## 2 Magnesium and magnesium alloys for biomedical applications

### 2.1 Applications of magnesium alloys for biomedical applications

The application of Mg alloys in biomedical field has a long history. Mg was first used as ligatures for bleeding vessels in 1878, and since then Mg alloys have been extensively studied in medical and surgical fields, including cardiovascular, musculoskeletal, and general surgery (Witter, 2010). Currently, the applications of biomedical Mg alloys mainly include cardiovascular stents and bone implants. Mg as a vascular stent is beneficial to regulate heart rhythm, improve blood flow, inhibit platelet activation, and prevent vasoconstriction (Banjanin and Belojevic, 2018). Moreover, Mg-based vascular stent can widen the narrowed arteries and maintain them until the vessel completes remodeling, then gradually degrades and is replaced by neovascular tissue (Liu et al., 2019b).

As an orthopedic implant, Mg alloy is a novel medical material, which can replace bone implants such as steel nails in traditional medicine, and better match the mechanical performances of human bone and avoid stress shielding effects than Ti alloys (Wang et al., 2008; Amukarimi and Mozafari, 2021). Furthermore, Mg alloys can be degraded into non-toxic and harmless small molecules after the human bones are basically healed, and are excreted through the human circulatory system, avoiding the pain of patients suffering from the second surgery to remove the implant. It has been reported that Mg as an orthopedic biomaterial promotes bone remodeling and healing (O’Neill et al., 2018; Zhou et al., 2021). In summary, Mg and its alloys are extremely valuable and potential biomaterials, especially for orthopedic applications.

### 2.2 Characteristics of magnesium alloys for biomedical applications

Mg alloys have been widely applied in biomedical field due to their high strength, low density, and good biocompatibility (Table 1). However, due to the high corrosion rate caused by the lowest standard electrode potential of Mg (−2.37 V), the excessively fast degradation rate of Mg alloys after implantation in the human body destroys the mechanical support before the reconstruction of damaged bone tissue (Cai et al., 2022). The disadvantages of Mg alloys for biomedical applications are listed in Table 2.

**TABLE 1:** Advantages of Mg alloys for biomedical applications.

Advantages	Description	References
Low density and elastic modulus	Density and elastic modulus are similar to those of cortical bone	Sezer et al. (2018)
High specific strength	The strength to weight ratio is approximately 35–260 kNm/kg	Seetharaman et al. (2022)
Machinability	Mg has excellent machinability, is easy to achieve stable dimensions and can be easily processed into complex shapes	Kirkland et al. (2011)
Stress shielding effect	The elastic modulus of Mg is very close to that of bone, many problems associated with implant stress shielding can be greatly reduced	Seetharaman et al. (2022)
Biocompatibility	Mg is biocompatible and has been shown to have osteogenic functions	Amukarimi and Mozafari, (2021)
Degradability	Mg eventually degrade completely in the body, which is beneficial to the patient	Kumar et al. (2018)

**TABLE 2:** Disadvantages of Mg alloys for biomedical applications.

Disadvantages	Description	References
Low mechanical properties	Implants generally need to be able to withstand a certain load and deformation. At present, it is difficult for most Mg alloys to meet clinical needs in both strength and plasticity	Seetharaman et al. (2022)
High degradation rate	It is easy to cause premature loss of mechanical integrity and support of implants, which restricts its application in clinical treatment, especially in orthopedic load-bearing parts	Ding, (2016)
Hydrogen (H_2_)	H_2_ released during Mg degradation accumulates in the surrounding soft tissue	Razavi and Huang, (2019)

Among them, the corrosion behavior of Mg alloy implants needs to be paid the most attention. When Mg is placed in an aqueous solution, Mg^2+^ cations are generated on the Mg surface due to the anodic reaction of Mg, as shown in Eq. 1 (Amukarimi and Mozafari, 2021). Meanwhile, a cathodic reaction occurs when protons are reduced at the cathode, producing H_2_ gas and OH^−^ ions (Eq. 2). Eventually, the Mg(OH)_2_ film covers the Mg surface (Eq. 3).

$$\text{Anodic reaction: Mg }\to {\text{ Mg}}^{2+}{+\;2\text{e}}^{-}$$ (1)

$${\text{Cathodic reaction: }2\text{H}}_{2}{\text{O }+\;2\text{e}}^{-}\;\to {2\text{OH}}^{-}{\;+\text{H}}_{2}$$ (2)

$${\text{Product formation: Mg }+\;2\text{H}}_{2}\text{O }\to \text{ Mg}{\left(\text{OH}\right)}_{2}{\;+\text{ H}}_{2}$$ (3)

**FIGURE 1:**
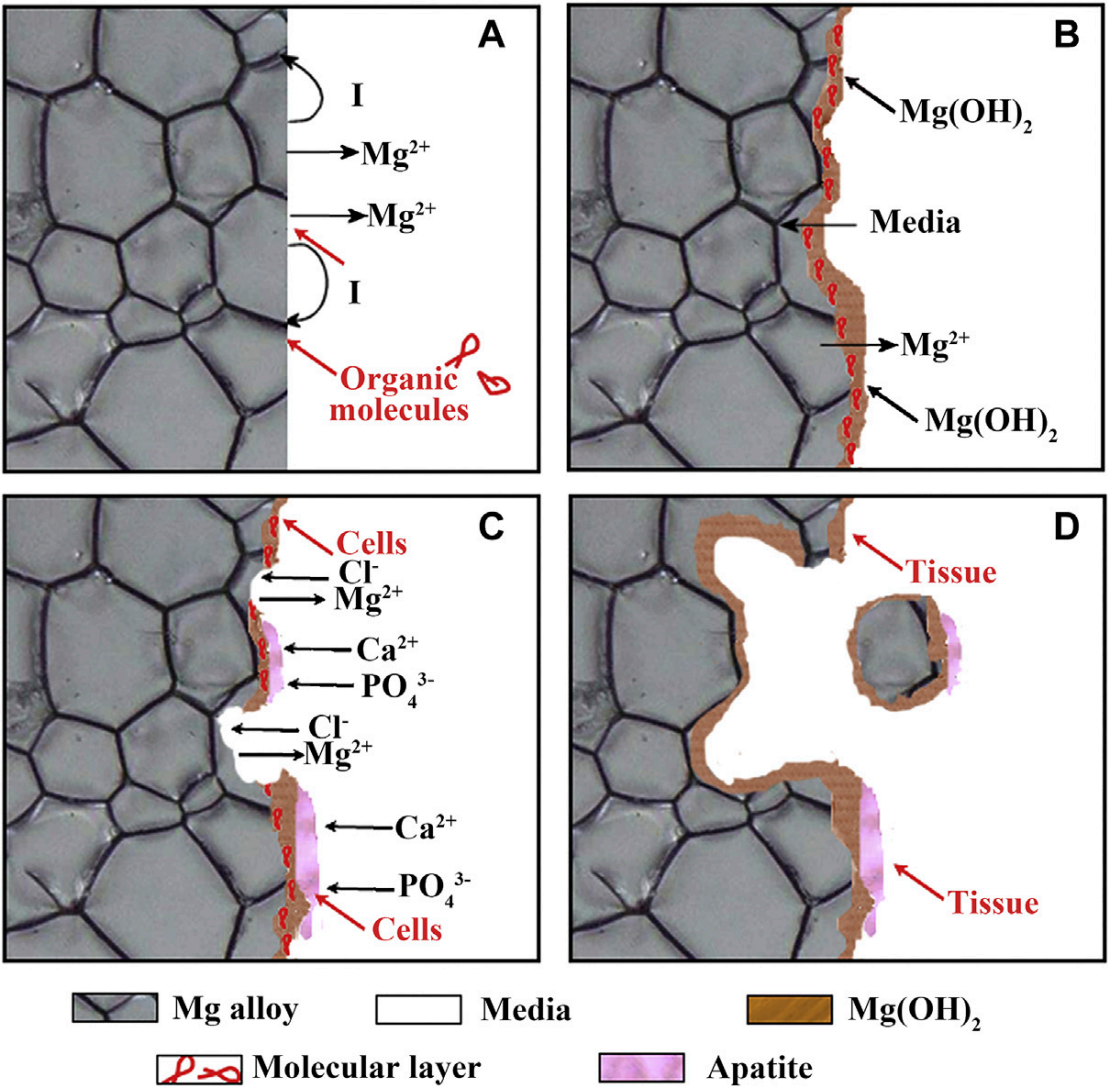
**(A–D)** Schematic illustration of the biocorrosion at the interface between Mg and medium. Reprinted with permission from reference (Zheng et al., 2014), Elsevier.

The corrosion behavior of Mg in the human body is more complex. Despite considerable efforts by researchers, it is still not fully understood. The corrosion degradation process of biomedical Mg alloys in body fluids can be shown in Figure 1. Electrochemical reactions (Eqs 1–4) occur arbitrarily across the surface, resulting in galvanic coupling because of different potentials between the Mg substrate and intermetallic phases or grain boundaries (Figure 1A). Moreover, some organic molecules may be adsorbed on the surface of Mg alloys, affecting the corrosion process of the material (Figures 1A,B). The OH^−^ ions generated during the reaction process will cause the local environment to be alkaline, resulting in a Mg(OH)_2_ film covering the Mg substrate surface, separating the Mg from the surrounding environment. However, the produced Mg(OH)_2_ film is loose and porous, and the external corrosion medium can further corrode the fresh Mg substrate through these holes, forming corrosion pits and producing a large amount of Mg(OH)_2_ (Amukarimi and Mozafari, 2021). It is worth noting that the human environment contains numerous chloride ions (Cl^−^), and when the Cl^−^ ions concentration reaches 30 mmol/L, it will lead to the conversion of Mg(OH)_2_ into soluble MgCl_2_ (Dong et al., 2022a). The concentration of Cl^−^ ions in the human body is about 150 mmol/L, and these Cl^−^ will damage the Mg(OH)_2_ layer and cause local corrosion defects (Figure 1C). The reactions as shown in Eqs 4, 5 (Seetharaman et al., 2022).

$${\text{Product formation: Mg}\left(\text{OH}\right)}_{2}{\;+\;2\text{Cl}}^{-}\to {\text{ MgCl}}_{2}$$ (4)

$${\text{Product formation: Mg}}^{2+}{\;+\;2\text{Cl}}^{-}\to {\text{ MgCl}}_{2}$$ (5)

As Mg(OH)_2_ layer is destroyed, the Mg substrate is further exposed, the local alkalinity increases, the Ca^2+^ and PO_4_^3−^ ions contained in the body fluid use the residual Mg(OH)_2_ as the nucleation sites to form calcium phosphate based apatite. Carbonates also be formed in the presence of carbonic acid or CO_2_, and these products co-deposit onto the surface of Mg substrate, forming a corrosion products layer. Cells are also found to adhere on the Mg surface. As the implantation time increased, the adhered cells proliferate, forming new tissues close to the corrosion product layer. In addition, corroded Mg may separate and fall off the substrate in the form of particles. The particles may be surrounded and swallowed by the fibrous tissue or macrophages until complete degradation (Figure 1D).

### 2.3 Factors affecting performances of magnesium alloys for biomedical applications

The service environment of biomedical Mg alloys is complicated, and its mechanical performances and corrosion behaviors mainly depend on alloy composition, microstructure, environmental medium as well as stress.

#### 2.3.1 Alloy composition

Despite the many advantages of Mg as a biomedical material, the application of pure Mg is limited because of its low corrosion resistance and insufficient mechanical performances. Although its corrosion in aqueous solution can be used for cathodic protection of batteries, corrosion resistance is required in structural applications. In addition, pure Mg shows low ductility and strength owing to the lack of the slip system inherent in the HCP structure, which can be improved by selecting appropriate alloying elements (Seetharaman et al., 2022).

Alloy composition can change the microstructure of Mg alloys, as well as the potential difference and surface potential between phases, thereby affecting mechanical performances and corrosion resistance. The alloying elements most commonly added to Mg are aluminum (Al) and zinc (Zn), since they increase the hardness, strength, and castability (Seetharaman et al., 2022). Lithium (Li) is a potential element for developing novel Mg alloys due to its low density and high solid solubility. It has been reported that the addition of Li more than 11.5% can change the crystal structure from HCP to BCC of Mg, thereby improving the formability (Pekguleryuz et al., 2013). In addition, alloy composition also affects the formation of oxide film or corrosion product film on the alloy surface. It was demonstrated that Mg-9.29Li-0.88Ca (Zeng et al., 2014) added with Li and Ca and Mg-4Zn-0.5Ca (Cho et al., 2017) added with Mn could form protective films in simulated body fluids, thus increasing the corrosion resistance of the alloys.

#### 2.3.2 Surface morphology

Since the surfaces of Mg alloys are in direct contact the surrounding environment in the human body, the surface conditions such as roughness and microstructure are also the main factors affecting their biomedical performances. Generally, the surface roughness plays a role on the corrosion behaviour of metallic materials. An increase in the surface roughness of stainless steels has been reported to increase the pitting susceptibility and corrosion rate (Amiriafshar et al., 2020). A similar trend occurred in copper (Xia et al., 2022) and Ti-based alloys (Pal et al., 2021). For Mg alloys, Xu et al. (2020) reported that the continuous protective film formed on alloys with smooth surfaces is higher than that on irregular surfaces, and the increase in surface roughness of Mg alloys affects the passivation tendency, thereby increasing the pitting susceptibility of the alloys.

Numerous researches have shown that the fine grain can enhance the mechanical performances and corrosion resistance of Mg alloys (Cui et al., 2021; Dobkowska et al., 2021; Klu et al., 2022). In particular, grain refinement promotes the increase of grain boundaries as corrosion barriers, which can more effectively block the expansion of pitting corrosion. The grains are refined and the microstructure is more uniform, and a more uniform and dense passivation layer can be formed (Ralston et al., 2010). Twining and texture also have obvious impact on the performances of Mg alloys. Liu et al. (2018a) carried out solution and then extrusion (T3) and solution (T4) treatments on EW75 alloy, and found that the T3 alloy with twinning structure showed better corrosion resistance. Luo et al. (2020) studied the corrosion behavior of Mg-6Gd-2Y-0.2Zr alloy and found that the (11 $\bar{2}$ 0) and (10 $\bar{1}$ 0) planes with higher atomic density were more prone to corrosion.

Besides, the second phase is also one of the key factors affecting the corrosion resistance of Mg alloys. The second phases with different types, shapes, and distributions will be produced by adding different types and contents of elements and using different processes. The most common influence mechanism is that the corrosion potential of the substrate is lower than that of the second phase, the two form a micro-battery, and the substrate acts as the anode to corrode first. However, the electrode potential of the second phase is lower does not mean that its corrosion rate is better (Zhang et al., 2020a). The corrosion of Mg alloys is also related to the morphology and distribution of the second phase. Jönsson et al. (2006) found that the potential difference in AZ91D reached 220 mV, but the corrosion rate was lower than that of pure Mg. Because a large amount of network second phases at the grain boundaries covers the substrate and act as a corrosion barrier to prevent the propagation of corrosion to the substrate, thereby reducing the corrosion rate.

#### 2.3.3 Environmental medium

The composition of physiological solutions affects the biological activity of Mg alloys. The main components that affect the corrosion of Mg alloys are inorganic ions, proteins, and cells. Jang et al. (2013) studied the effect of different inorganic ions on the degradation of Mg alloys in the human environment. The research showed: 1) When there were only two anions of Cl^−^ and OH^−^ in the environment, the corrosion product layer does not contain calcium; 2) When HPO_4_^2−^ and Cl^−^ co-exist in solution, the phosphate could induce the formation of a dense amorphous magnesium phosphate corrosion product layer; 3) When HPO_4_^2−^ and Ca^2+^ existed together in NaC1 solution, octacalcium phosphate, and hydroxyapatite layers would be formed on the Mg alloy surface, which could inhibit local corrosion and improve overall corrosion resistance; 4) The degradation rate was accelerated by HCO_3_^−^ ions.

In addition to inorganic ions, there are various proteins in human plasma. The research results show that the effect of protein on the corrosion rate of Mg alloys is related to the alloy type, time, and concentration (Virtanen, 2011). Although albumin has little effect on the electrochemical behavior of AZ31 alloy, it greatly promotes the anodic dissolution of pure Mg and LAE442 alloy (Mueller et al., 2009). When the AZ31 alloy is exposed to serum protein, serum protein increases the corrosion rate of the substrate within the first 3 days (Gu et al., 2009). Although the protein adsorption layer generally acts as a barrier between the metal surface and the surrounding environment, hindering corrosion to a certain extent (Yang et al., 2012), this protein adsorption layer is not very dense. Moreover, the performances of the protein adsorption layer can change dramatically over time due to Mg corrosion, for example, high pH on the Mg surface can lead to protein denaturation or even exfoliation.

The mechanism of the effect of cells on the corrosion of Mg alloys *in vitro* is not very clear. The adhered cell layer acts as a barrier to slow down the corrosion process (Seuss et al., 2011). However, Zhang et al. (2014) demonstrated that human umbilical vein endothelial cells can accelerate the corrosion rate of Mg, which may be due to the process of cell metabolism and proliferation that promotes protein desorption and storage of Mg^2+^ ions in the medium.

#### 2.3.4 Stress conditions

As a biomedical material, after being implanted into the human body, Mg faces different stress conditions at different implant sites, which also affects the corrosion of the substrate. For example, after the Mg alloy vascular stent is implanted into the human body, it is exposed to blood fluid in the initial stage, and the stent is mainly subjected to the shear force of blood flow. In the subsequent stage of tissue coating the stent, the growth of the intima on the surface of the stent is again influenced by fluid diffusion. Fluid flow has a major effect on the degradation of Mg scaffolds, which can increase the overall corrosion rate of the implant due to the presence of shear stress (Wang et al., 2014; Saad et al., 2017). Simultaneously, the thickness of the corrosion layer, the area and depth of localized corrosion and the exfoliation of corrosion products in the corrosion pit also further expanded by shear stress (Wang et al., 2014).

## 3 Modification of magnesium alloys for biomedical applications

Although Mg has the advantages of mechanical performances close to bone and the best biocompatibility among biodegradable metals (Chen et al., 2018; Qin et al., 2019), the high corrosion rate in the human body greatly hinders its development and application. Therefore, improving the corrosion resistance of Mg is the key to overcome the above-mentioned drawbacks in biomedical applications. There are two methods for controlling the corrosion behavior of Mg alloys. One is to tun the composition of Mg alloys through high purification or alloying, and the other is to change the microstructure of metal surface or form a protective coating on the surface through surface treatment (Rendon et al., 2019; Amukarimi and Mozafari, 2021). Recently, researchers have made a lot of efforts to achieve optimal corrosion resistance of Mg biomaterials by devising novel Mg alloys and surface modification techniques.

### 3.1 Optimization of alloy composition

#### 3.1.1 Purification

The corrosion of Mg alloys is related to the content of impurity elements (Cao et al., 2013). Commercially pure Mg often contains a large amount of impurity elements (Fe, Ni, Cu, etc.), the corrosion potential of these elements is much higher than that of Mg, which is easy to cause galvanic corrosion, thereby accelerating the dissolution of Mg and the hydrogen evolution reaction (Atrens et al., 2018). Therefore, it is possible to reduce the corrosion rate and enhance the mechanical performances by improving the purity of biomedical Mg alloys and controlling the content of harmful elements to keep them at the allowable limit concentration (Yamamoto and Hiromoto, 2009). At present, the main method to improve the purity is by selecting high-purity raw materials, optimizing the smelting process and adding Mn, Zr, and other elements to reduce impurities (Prasad et al., 2013). Although the degradation of Mg alloys can be effectively slowed down by purifying, the corresponding mechanical performances are reduced while impurities are removed, which limits its further application (Qiao et al., 2012).

#### 3.1.2 Alloying

Alloying is one of the main ways to advance the mechanical performances of metals (Chen et al., 2015; Wei et al., 2022). In particular, the addition of appropriate alloying elements can refine grains, optimize the type and size as well as distribution of second phase, thereby enhancing the corrosion resistance of Mg alloys. Moreover, elements can form passive films or corrosion product layers to inhibit the further expansion of corrosion. Currently, aluminum (Al), zinc (Zn), manganese (Mn), calcium (Ca), strontium (Sr), strontium (Zr), neodymium (Nd) elements are widely used as alloying elements. The effects of these elements on performances of Mg alloys are shown in Table 3.

**TABLE 3:** Effects of common alloying elements on performances of Mg alloys.

Alloying elements	Biocompatibility	Corrosion resistance	Mechanical performances	References
Al	Ai is neurotoxic, it may cause Alzheimer’s disease and damages muscle fibers	It is beneficial for corrosion resistance	The addition of Al increases the strength and plasticity	Agarwal et al. 2016; Mirza et al. 2017
Zn	Zn is an essential trace element for human body, with non-cytotoxic and good biocompatibility	It makes the corrosion resistance decreases with the increase of Zn content	Zn mainly plays the role of solid solution strengthening, and the strength increases with the increase of Zn content	Tian et al. 2016; Becerra et al. 2020
Mn	Mn is an essential trace element for human body. However, it has been reported that Mn is cytotoxic and neurotoxic	It is beneficial for the corrosion resistance	It increases yield strength, and decreases tensile strength and elongation	Cho et al. 2017; Liu et al. 2021b
Ca	Ca is an important component of human bone, with non-cytotoxic	It makes the corrosion resistance reduces with increasing Ca content	It makes the strength increases and plasticity reduces with increasing Ca content	Zhang et al. 2017b; Mohamed et al. 2019
Sr	Sr is an important component of human bone, with non-cytotoxic. It can promote bone formation	It makes the corrosion resistance of Mg alloys reduces with increasing Sr content	It makes the strength increases with increasing Sr conten.t	Dong et al. (2022a)
Zr	Zr has good biocompatibility and bone bonding ability	It makes the corrosion resistance reduces with increasing Zr content	It makes the grains refine, strength and plasticity increase	Agarwal et al. 2016; Zhou et al. 2020a
Si	Si is an essential trace element for human body	It reduces the corrosion resistance	It produces coarse Mg_2_Si phase, increases strength and decreases plasticity	Qin et al. (2019)
Li	Li may cause malformation of human cardiovascular system	It reduces the corrosion resistance	When the addition amount of Li exceeds 5.5%, the microstructure changes, the strength decreases and the plasticity increases	Wu et al. 2017; Kumar et al. 2018
Nd	Nd is cytotoxic at high concentration, while it has good biosafety at low concentration	It improves the corrosion resistance	It makes the new phases form, microstructure refine, mechanical performances improve	Xie et al. (2021)
Y	Y has good biocompatibility	It improves the corrosion resistance	It increases the strength and plasticity	Ren et al. 2017; Nie et al. 2021
Ce	Ce is high cytotoxic	It improves the corrosion resistance, while more amount reduces corrosion resistance	It improves the strength and fatigue resistance	Amukarimi and Mozafari (2021)
La	La is higher cytotoxic	It improves the corrosion resistance	It improves the strength and creep resistance	Ding et al. (2014)
Er	Er is cytotoxic	It improves the corrosion resistance	It improves strength and plasticity	Zhang et al. 2010; Zhang et al. 2012a
Gd	Gd is cytotoxic	It improves corrosion resistance, which decreases when Gd content is high	It improves strength due to solid solution strengthening	Chen et al. (2019b)

Al is the most added element in commercial Mg alloys, which can not only refine the grains, but also improve the corrosion resistance (Mirza et al., 2017). After adding Al, the Al-rich layer, and β-phase (Mg_17_Al_12_) network formed on the corrosion surface can effectually prevent further corrosion of Mg alloys. Many Mg alloys containing Al element, such as AE21, AZ31, and AZ91, have been used in biomedical fields due to their good mechanical performances and corrosion resistance. However, Al is considered to be neurotoxic and may cause Alzheimer’s disease (Agarwal et al., 2016; Mirza et al., 2017).

Zn, as an essential trace element for the human body, can also effectively improve the mechanical performances of materials (Becerra et al., 2020), which is usually added in Mg-Al alloys. As one of the main research interests of novel medical materials, Mg-Zn alloys exhibit good biocompatibility (Bîrcă et al., 2018). The addition of Zn has a great effect on the corrosion properties of Mg alloys. For instance, Koç et al. (2015) reported that in Mg-Zn alloys, the increase of Zn content resulted in grain refinement, the formation of passivation films, and the formation of Zn oxide layers and the precipitation of eutectic phases, which significantly slowed down the degradation rate. Yan et al. (2017) investigated the microstructure and corrosion behavior of Mg-6Zn, Mg-14.5Zn, Mg-25.3Zn, and Mg-40.3Zn (wt.%) alloys. They found that the MgZn phase was the dominant intermetallic phase when Zn was added at 6 and 14.5 wt.%, respectively. When the addition of Zn reached 25.3 and 40.3 wt.%, a large number of MgZn_2_ phases and Zn particles were formed. The higher the Zn concentration, the larger the intermetallic phase. The more Zn particles, the more serious the microgalvanic corrosion. The concentration of Zn has a great impact on biomedical effects of Mg alloys, but there is still no systematic study to define the concentration limit of Zn in biodegradable Mg alloys (Tian et al., 2016). Therefore, more research on the content limitation of Zn is required in the future to improve the clinical application of Mg-Zn alloys.

Mn is one of the essential trace elements for the human body. It can refine the grains, and can convert the impurity elements such as Fe, Ni into intermetallic compounds to precipitate, and may also form a Mn-containing oxide film to prevent the infiltration of Cl^−^ ions, thus it is widely used in biomedical Mg alloys (Cho et al., 2017; Liu et al., 2021b). Liu et al. (2021c) a added Mn to as-extruded Mg-0.5Bi-0.5Sn alloys, and demonstrated that the addition of Mn resulted in a decrease in the average grain size, and the corrosion rate of the alloy reduced from 0.59 mm/a to 0.22 mm/a when only 0.5% Mn was added in the simulated body fluid. Compared with the alloy without Mn element, the alloy exhibited a more uniform corrosion morphology, which provided favorable conditions for the dynamic balance between the formation and destruction of the corrosion product film, and hindered the further corrosion of Mg substrate. Rosalbino et al. (2010) studied the corrosion resistance of Mg-2Zn-0.2X (X = Ca, Mn, Si) alloys in simulated body fluids, and the results showed that Mg-2Zn-0.2Mn had better corrosion resistance. However, the cytotoxicity and neurotoxicity of Mn have been reported (Ding et al., 2011; Zheng et al., 2014). Mn damages the sensory epithelial cells and auditory nerves of organisms, and causes severe lesions in neurons and hair cells.

Ca is an essential element for human body and an important component of bone, the density is close to that of human bone (Ding et al., 2014), which makes Mg-Ca alloy exhibit greater advantageous as a bone implant material. The addition of Ca with an appropriate amount can refine grains, inhibit grain boundary compounds, reduce the potential difference between the second phase and the substrate, and improve the density of oxide film, thereby hindering corrosion and increasing the corrosion resistance of Mg alloys (Zhang et al., 2017b; Mohamed et al., 2019). Zheng et al. (2010) prepared Mg-Ca alloys with 1 ∼10 wt.% Ca content and analyzed their corrosion resistant. Electrochemical tests and *in vitro* simulation tests showed that the corrosion resistance of Mg-Ca alloys with 5 wt.% and 10 wt.% Ca content was significantly reduced compared to 1 wt.% Ca. Zhang et al. (2011) found that the addition of Ca (0.2 wt.%) decreased the degradation rate of as-cast Mg-4Zn alloy (∼30%), because Ca reduced the potential difference between the second phase and the substrate. Studies have shown that for Mg-Ca alloys, increasing the Ca content is beneficial to increase the Mg_2_Ca phase as well as the compressive strength, elastic modulus, and hardness, but reduce the plasticity, corrosion resistance, and biocompatibility (Li et al., 2011; Agarwal et al., 2016). It is worth noting that the mechanical performances, corrosion resistance, and biocompatibility of the alloy are better when the Ca content is low (less than 1 wt.%) (Li et al., 2011).

Sr is one of the trace elements in the human body. Almost all of the Sr exists in the bones. Strontium salts can promote the formation of bones (Bornapour et al., 2013). The chemical performances of Sr, Mg, and Ca are similar. The addition of Sr to Mg alloys can effectively refine grains and improve comprehensive properties. Therefore, Sr elements are often added to Mg alloys for bone implantation in recent years. Mg-Sr alloys also exhibit good biocompatibility. For instance, Ragamouni et al. (2013) indicated that Mg-Zr alloy with Sr addition fuses better with new bone tissue in bone tissue of rabbit.

In addition, Zr, Si, Li elements are often added to Mg alloys for biomedical applications. The good biocompatibility and bone bonding ability of Zr element have been reported (Agarwal et al., 2016; Zhou et al., 2020a). As an alloying element, Zr can refine grains and effectually increase the corrosion resistance of Mg alloys (Xing et al., 2021). Si is also one of the essential trace elements for the human body. It is often added together with other elements to improve the performances of Mg alloys, because the Mg_2_Si phase produced in Mg-Si binary alloys greatly reduces the ductility and corrosion resistance (Qin et al., 2019). Although Li may cause malformation of the human cardiovascular system, Mg-Li-based alloys exhibit good ductility, which can fulfill the requirements of expandable vascular stents (Kumar et al., 2018). Moreover, observed that Mg-Li-Zn ternary alloys showed good biocompatibility.

Low alloying of rare Earths is a direction to develop high-performance Mg alloys. The addition of a small amount of rare Earth elements can significantly affect the microstructure and performances of Mg alloys. At present, rare Earth elements commonly added in biomedical Mg alloy include Nd (Xie et al., 2021), Y (Ren et al., 2017; Nie et al., 2021), Ce (Amukarimi and Mozafari, 2021), La (Ding et al., 2014), Er (Zhang et al., 2010; Zhang et al., 2012a), Gd (Chen et al., 2019b; Luo et al., 2020), etc. Nd can form new phases with Mg and other chemical elements in Mg alloys, thereby refining the microstructure and improving the mechanical performances of the alloys (Xu et al., 2014). In particular, Zhang et al. (2012b) and Xie et al. (2021) reported that the Mg-Nd-Zn-Zr alloys developed by adding elements such as Nd exhibit good mechanical performances ( > 200 MPa), corrosion resistance (corrosion rate is ∼0.125 mm/a), and better biocompatibility. The addition of Y improves the corrosion resistance of Mg alloys. Ren et al. (2017) observed that when the Y content is less than 1%, the corrosion resistance of the alloy increases with the increase of the Y content due to the grains refinement. However, too much Y added to the alloy will lead to the formation of the MgY phase, forming many micro-batteries and accelerating the corrosion of the alloy. Although Ce, La, and Er have high cytotoxicity among rare Earth elements (Feyerabend et al., 2010; Zhang et al., 2012a), the addition of a small amount of above elements to Mg alloys will not damage human body, and can significantly improve mechanical performances and corrosion resistance (Zhang et al., 2010). Chen et al. (2019b) observed that when a low content of Gd (0%–1%) was added to the Mg-2Zn-0.5Zr alloy, the second phase was uniformly distributed, and the addition of Gd stabilized the degradation layer and reduced the degradation rate of the alloy. However, when the addition of Gd was increased to 2%, large and thick second phases formed along the grain boundaries, which increased the alloy corrosion rate due to electrochemical corrosion.

With the in-depth study of alloying, the mechanism of improving the corrosion resistance of Mg alloys by alloying has gradually become clear. Only a small amount of alloying elements are added to biomedical Mg alloys to obtain great performances, and excessive elements will deteriorate the corrosion properties. Moreover, the more elements added, the more uncertain factors in the performance determination of medical Mg alloys, and the greater challenges in biosafety assessment. Therefore, the comprehensive properties of biomedical Mg alloys including corrosion degradation, mechanical performances, and biosafety should be comprehensively considered to add appropriate alloying element.

### 3.2 Surface modification

In addition to changing the corrosion resistance of Mg alloys, surface modification is also an important means to advance their corrosion resistance. Appropriate surface modification methods can not only enhance the mechanical performances and corrosion resistance of Mg alloys, but also simultaneously improve the biological function of the alloys such as biocompatibility and bioactivity (Yu et al., 2018; Wu et al., 2019). Currently, a variety of surface modification technologies have been developed for biomedical Mg alloys, which can be divided into surface coating preparation and surface microstructure modification.

#### 3.2.1 Surface coating preparation

Since the oxide film formed on the Mg surface is relatively loose, it cannot protect the alloy for a long time. Therefore, it is effective to prepare a protective layer on the Mg surface by chemical, physical, mechanical, and biological or biomimetic techniques (Yin et al., 2020).

##### 3.2.1.1 Chemical conversion coatings

Chemical conversion coatings are formed by the electrochemical or chemical reaction of Mg-based materials, and the bath usually includes fluoride, phosphates, carbonate, and chromate (Wandelt, 2018; Rahim et al., 2022). An insoluble compound film with good adhesion can be formed on the Mg surface by chemical conversion treatment, which can not only protect Mg alloy from water and other corrosive environments, but also improve the adhesion of subsequent coatings. The method is easy to operate, which is widely used for biomedical applications. Among them, fluoride coatings (Pereda et al., 2011) and phosphates coatings (Chen et al., 2014a; Willbold et al., 2015) for biomedical Mg alloy surfaces have attracted extensive attention.

Typically, fluoride conversion coatings are performed in hydrofluoric acid (HF) solutions by chemical reactions with Mg alloys (Lin et al., 2013; Yan et al., 2014). The main component of the fluorine conversion coating is magnesium fluoride (MgF_2_), which is insoluble in water and easily deposited on the Mg surface. MgF_2_ films have been used in biomedical Mg alloys due to their good corrosion resistance, improved cellular response, and biocompatibility (Liu et al., 2015b). Barajas et al. (2019) prepared a magnesium hydroxyfluoride coating on the AZ31 alloy surface using 4% and 10% HF solution respectively. The corrosion current density of the fluorinated samples is reduced by about three orders of magnitude, which improves the corrosion resistance of the alloy and biocompatibility. Furthermore, Fintová et al. (2019) fabricated fluoride conversion coatings though immersing AZ61 Mg alloy into sodium fluoroborate [Na (BF_4_)] molten salt under different temperature, and then boiling in distilled water to remove the residual salts and the outer layer. The obtained coating exhibited a double layer structure with a thick inner Mg-F (MgF_2_) layer and a thin outer Na-Mg-F (NaMgF_3_) layer. The corrosion current density i_corr_ of the coatings decreased with increasing treatment time, indicating an increase in corrosion resistance in SBF solution. Therefore, fluoride conversion coatings are a good way for improving the corrosion resistance of Mg alloys.

**FIGURE 2:**
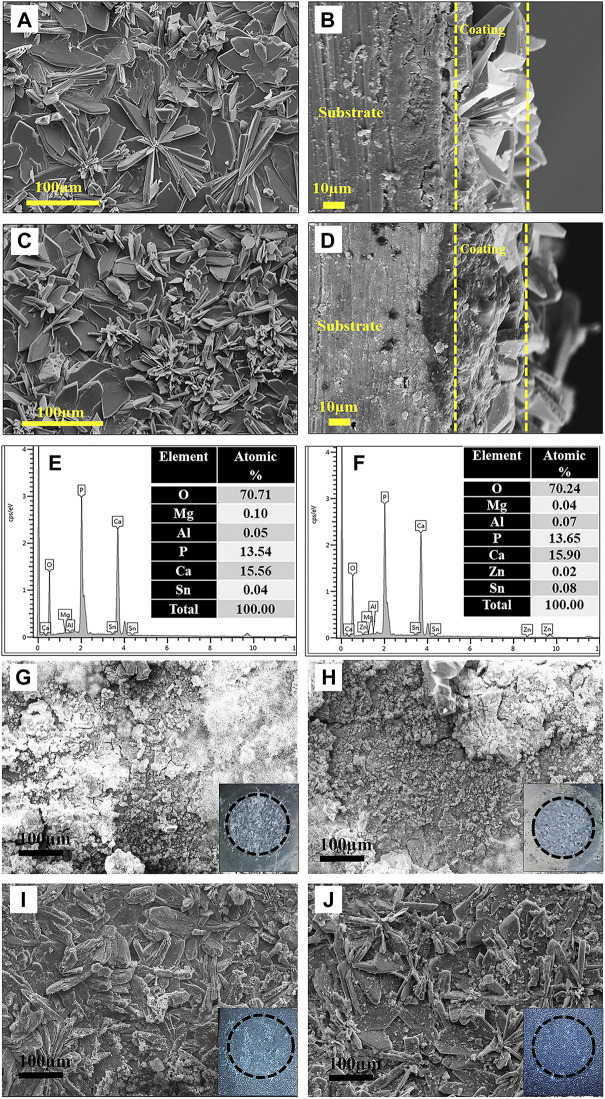
SEM images: **(A,C)** PCC coated surface and **(B,D)** cross-section of LAT971 and LATZ9531; EDS spectrum: **(E)** LAT971 and **(F)** LATZ9531 alloy; SEM images after corrosion test: **(G,H)** uncoated and **(I,J)** PCC coated LAT971 and LAT9531, respectively. Reprinted with permission from reference (Maurya et al., 2018), Elsevier.

Recently, phosphate conversion coatings such as zinc phosphate (Zeng et al., 2011), calcium phosphate (Chen et al., 2014b) have been reported due to their water insolubility, high temperature resistance, corrosion resistance, and great biocompatibility. For instance, Zeng et al. (2011) prepared two phosphate conversion coatings on the surface of AZ31 alloy, both of which showed higher corrosion resistance than the substrate. The flower-like Zn-Ca coating was denser than the rod-like Zn coating, which significantly reduced the corrosion current density. Besides, Maurya et al. (2018) improved the corrosion resistance of Mg-9Li-7Al-1Sn (LAT971) and Mg-9Li-5Al-3Sn-1Zn (LAT9531) by preparing the phosphate chemical conversion coatings. The microstructure of phosphate chemical conversion (PCC) coatings and the corrosion morphologies are shown in Figure 2. Since the coating could slow down the generation of degradation productions and hinder the occurrence of pitting corrosion, the degradation rates of coated-LA971/LAT9351 was reduced.

In summary, an insoluble compound film with good adhesion can be formed on the Mg surface by chemical conversion treatment, which can protect Mg alloy from water and other corrosive environments. In the future, chemical conversion coatings should be combined with other surface modification techniques to produce biofunctional coatings with excellent mechanical properties.

##### 3.2.1.2 Biomimetic deposition

**FIGURE 3:**
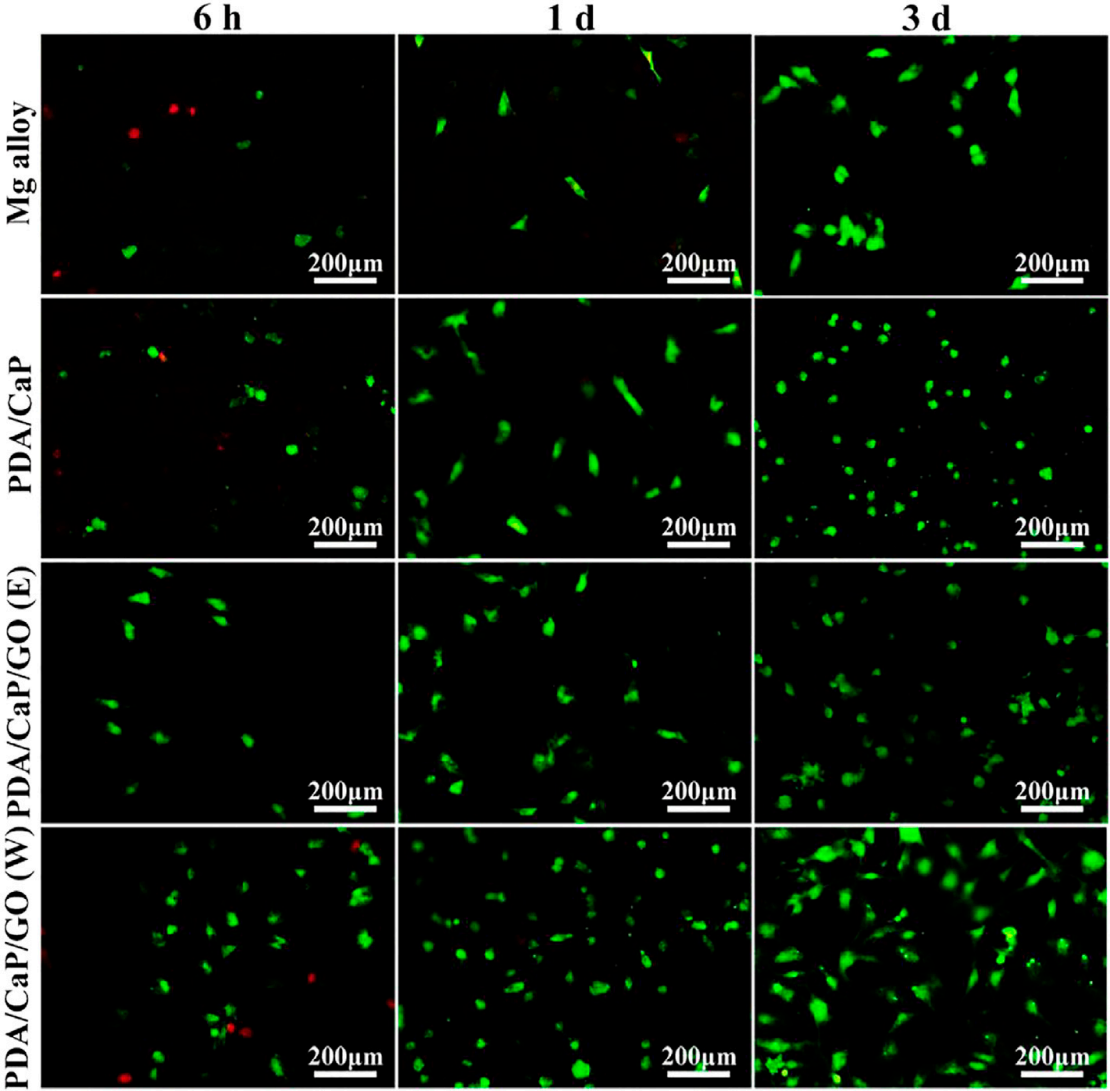
Fluorescence images of live/dead staining of cells after cultured on the different samples. Reprinted with permission from reference (Dong et al., 2022b), Elsevier.

Biomimetic deposition is a method developed in recent years to simulate the process of physiological apatite mineralization in nature and spontaneously deposit bioceramic membranes on the surface of substrates. The advantages of the biomimetic technique are as follows (Lin et al., 2015): First, the coating composition, phase, and crystallinity are easy to adjust; Second, the method can also produce biomimetic apatite coatings on porous or complex-shaped implants; Third, it is a simple and effective method of incorporating biologically active agents or drugs into apatite coatings through coprecipitation rather than *via* mere adsorption on the surface. Therefore, biomimetic method has been extensively applied for the modification of metallic biomaterials. For example, Hernandez et al. (2022) successfully deposited bioactive hydroxyapatite (HAp) coatings on pure Mg surfaces based on immersion of Mg substrates in supersaturated calcification solution (SCS). Gao et al. (2015) used biomimetic method to prepare bioactive hydroxyapatite/graphene oxide (HA/GO) hybrid coatings on Mg alloys, and they found that the formed HA/GO coating could significantly improve the corrosion resistance. Dong et al. (2022b) prepared polydopamine (PDA)-based calcium phosphate (CaP)/graphene oxide (GO) composite coatings on AZ60 Mg alloy. First, PDA was used as a pretreatment layer to induce the biomimetic deposition of CaP, and then GO as a sealing layer was spin-coated with ethanol and water as dispersants, respectively. Electrochemical and immersion test results showed that the corrosion resistance of the PDA/CaP/GO composite coating was significantly increased. The results of *in vitro* cell experiments showed that the composite coating could promote cell adhesion and improve biocompatibility (Figure 3). In addition, the corrosion resistance and biocompatibility of the PDA/CaP/GO composite coating with water as dispersant were better than those with ethanol, which was significantly improved compared with the Mg alloy.

In summary, the preparation of bioactive coatings on the surface of Mg and Mg alloys by biomimetic deposition can effectively improve the corrosion resistance and biocompatibility. However, the technology is still in the experimental stage at present, and there are few material systems that can be deposited. In the future, In-depth research should be carried out to promote the development of this technology.

##### 3.2.1.3 Micro-arc oxidation coating

**FIGURE 4:**
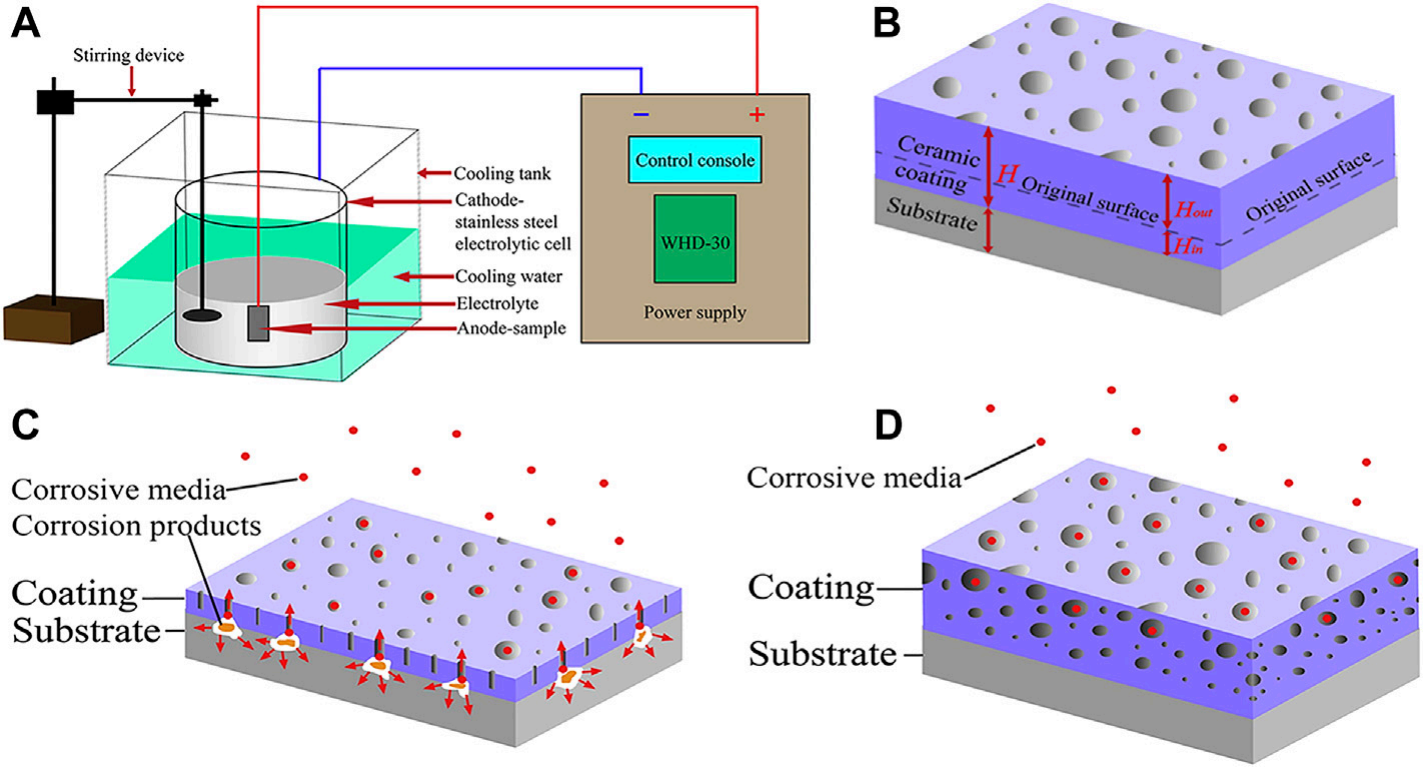
The schematic diagrams of **(A)** MAO device, **(B)** growth model; the corrosion mechanism of MAO coating: **(C)** thin coating with through-pores; **(D)** thicker coating with complex pores. Reprinted with permission from reference (Liu et al., 2020a), Elsevier.

Micro-arc oxidation (MAO), also known as plasma electrolytic oxidation (PEO), is a high voltage plasma-assisted anodic oxidation process developed from traditional anodizing to form ceramic-like coatings (Lu et al., 2016). The schematic diagram is indicated in Figure 4. MAO has been widely investigated innumerous fields because of its high efficiency, high bonding strength between coating and substrate, no limitation on the surface shape of the workpiece and so on (Liu et al., 2019a; Liu et al., 2020a).

The microstructure and performances of the MAO coating on the surfaces of biomedical Mg alloys have been extensively investigated. Liu et al. (2019a) observed that the prepared porous ceramic coating consists of dense inner and porous outer layers. Generally, a lower voltage produces a fine porous structure, while the pore dimension increases with treatment time and voltage. Therefore, a large number of micropores or high porosity is the main drawback for MAO coatings to realize long-term surface protection (Lu et al., 2016). Wang et al. (2020d) fabricated a dense MAO coating through a two-step current decreasing mode, which decreased the corrosion rate of material from 0.9690 to 0.1559 g/m^2^h in NaCl solution. Lin et al. (2021) prepared Li-added MAO coatings on pure Mg surface. The results showed that the corrosion resistance of the coated alloy was significantly higher than that of pure Mg, and the addition of Li reduced the number of micropores and cracks on the MAO coating, which made the alloy to exhibit better corrosion resistance. Furthermore, Liu et al. (2020a) prepared a protective MAO coating on the surface of Mg-based composite to reduce their degradation rate. They found that the corrosion resistance of the composite was significantly improved by the coating, and the corrosion resistance of the coating increased with oxidation time.

**FIGURE 5:**
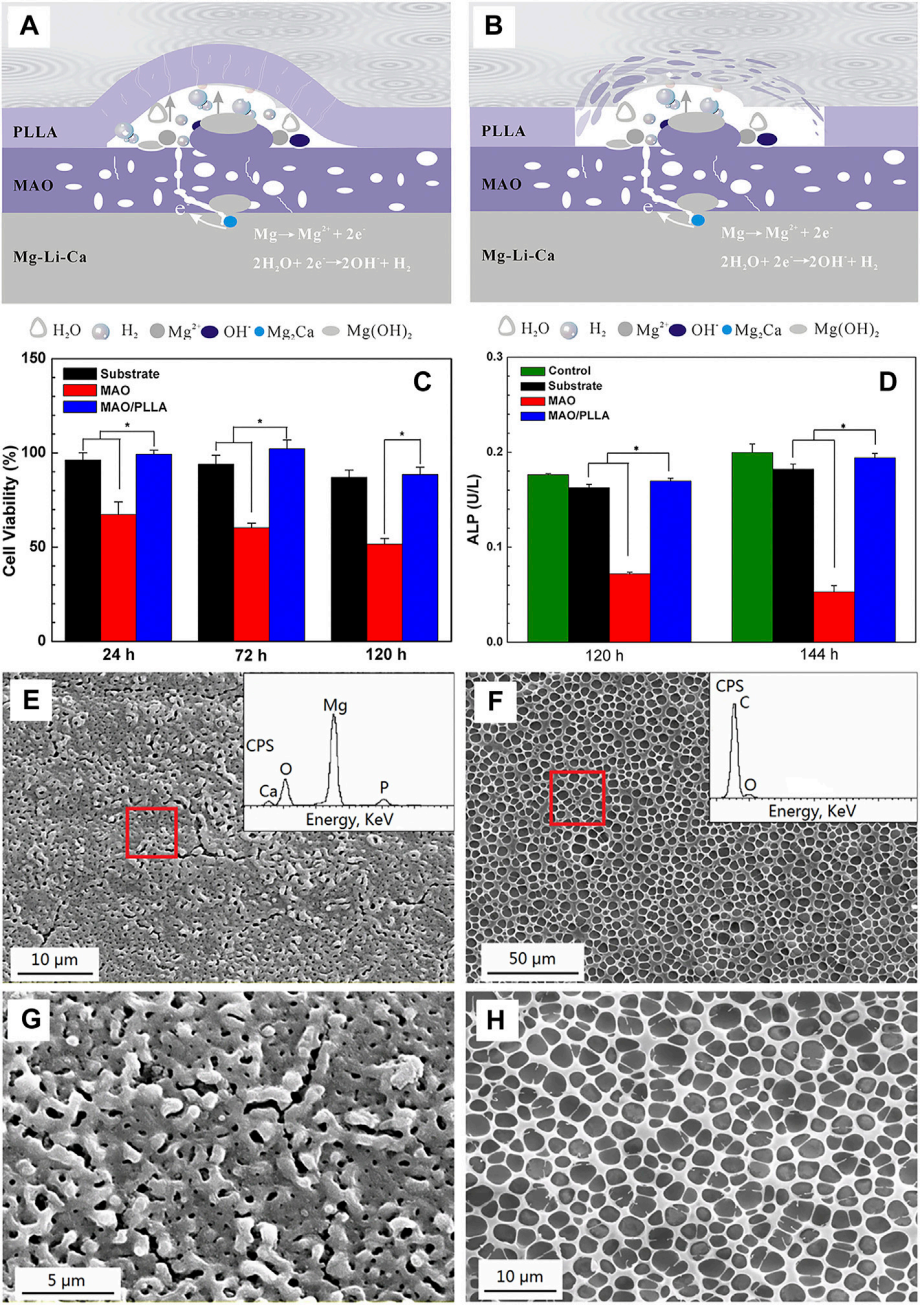
Schematic diagrams of the degradation mechanism of porous MAO/PLLA composite coatings on Mg-1Li-1Ca alloys: **(A)** swelling of PLLA and corrosion of the substrate, and **(B)** blistering and final peeling-off of PLLA; **(C)** RGR and **(D)** proliferation and differentiation of MC3T3-E1 cells cultured for different times in different extracts. Error bars represent ± S for *n* = 5 and 3, respectively, and *p* < 0.05, as indicated by the asterisk (*); SEM images of **(E)** the MAO coatings; **(F)** the MAO/PLLA composite coatings, **(G,H)** the high magnification view of e and f, respectively. Reprinted with permission from reference (Zeng et al., 2016), American Chemical Society.

For biocompatibility and biological activity, the MAO coating exhibits high bonding strength with the substrate due to the dense interior, and the porous outer layer is helpful to protein adsorption, osteoblast adhesion, and bone tissue regeneration, which is potential for biological applications. Wei et al. (2015) obtained a dense PEO/PLLA composite coating by sealing PEO with PLLA on AZ31Mg alloy. The results from *in vitro* tests showed that the degradation kinetics were obviously reduced, the hemolysis ratio was as low as 0.80%, and MC3T3-E1 cells displayed good adhesion and proliferation ability on the coating. Zeng et al. (2016) investigated the effects of MAO/PLA composite coatings on the corrosive behavior of Mg-1Li-1Ca alloys. They found that the MAO coatings could only provide limited protection to the substrate. Although it could protect the substrate during the initial immersion stage, the difference of free corrosion potential between the coating and substrate caused galvanic corrosion due to the presence of pores and microcracks during the subsequent immersion. Moreover, the top layer of PLLA on the MAO coating experienced swelling and subsequent delamination or peeling off under the pressure of H_2_ gas and corrosion products (Figures 5A,B). The results of biocompatibility test using fresh rabbit arterial blood showed that the hemolysis ratio decreased from 61.35% to 0.17% for the MAO/PLLA composite coated on Mg-Li-Ca alloy (Figures 5C,D). The composite coating with porous microstructure (Figures 5E–H) also promoted the attachment of MC3T3-E1 cells.

In summary, the bioactive coating prepared by micro-arc oxidation is beneficial to protein adsorption, osteoblast adhesion, and bone tissue regeneration. In the future, micro-arc oxidation technology should be developed for multilayered micro-nanostructured coatings for cell adhesion and proliferation, and to construct multifunctional coatings with biological activity and antibacterial properties.

##### 3.2.1.4 Sol-gel coating

**FIGURE 6:**
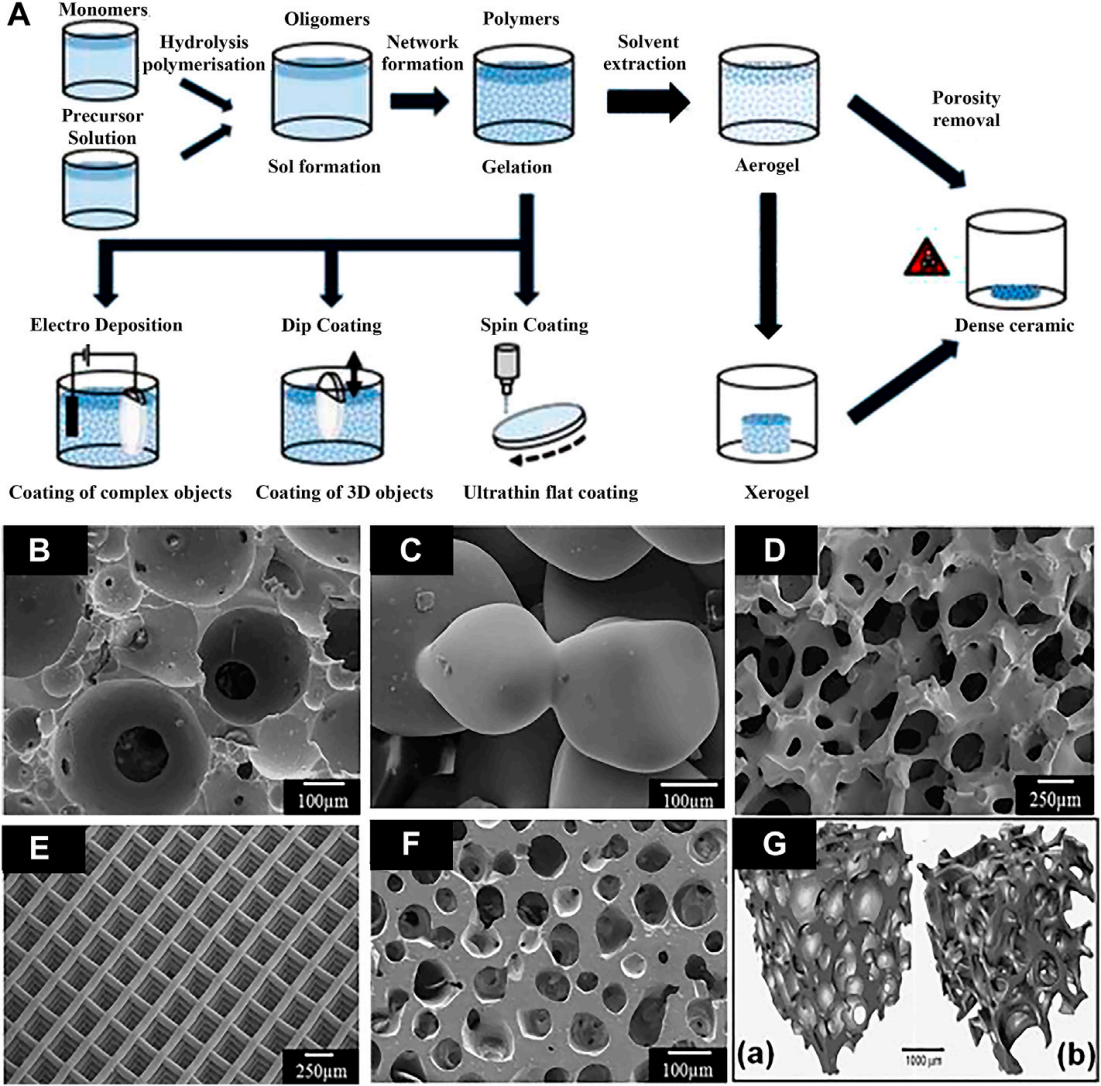
**(A)** Schematic illustration of preparation processes of the sol-gel coatings; **(B–F)** bioactive glass scaffolds with porous structure produced by sol-gel methods, and **(G)** micro-computed tomography image of typical scaffold and human trabecular bone. Reprinted with permission from reference (Owens et al., 2016), Elsevier.

The sol-gel process, also known as chemical solution deposition, has been extensively applied in materials science and ceramic engineering. The method is mainly used for synthesis of materials starting from a chemical solution that acts as the precursor for an integrated network (or gel) of either discrete particles or network polymers (Figueira et al., 2016). In general, sol-gel formation follows four steps: 1) hydrolysis, 2) condensation and polymerization of monomers to form chains and particles, 3) particle growth, and 4) agglomeration of the polymer structures followed by the network formation throughout in liquid medium which increases the viscosity to form a gel (Owens et al., 2016). The schematic illustration of preparation process of the sol-gel coatings is show in Figure 6A. The method has the advantages of low cost, low processing temperature, and the ability to coat a variety of materials into complex shapes, which is favorable for biomedical applications (Figueira, 2020). The porous scaffolds of a few bioglasses (BGs) has been prepared by the method, such as the glass designated 58S [60 SiO_2_-36 CaO-4 P_2_O_5_ (mol%)] by Sepulveda et al. (2002). The scaffolds with porous structures made of bioactive glasses produced by sol-gel processes in recent years are displayed in Figures 6B–F.

Combining the changes in the coating preparation process and adding other functional components, different functional coatings can be obtained. In addition, how to shorten the processing time and improve the bonding between the surface and the substrate by changing the processing parameters seems to be the main research direction in the future.

The microstructure and corrosion resistance of the sol-gel coatings have been investigated. Kania et al. (2020) observed that the 300 nm thick TiO_2_ films with an anatase structure were deposited on MgCa4Zn1Gd1 alloy by magnetron sputtering and spin coating methods respectively. Compared to the film prepared by spin coating, the surface of TiO_2_ film prepared using the magnetron sputtering method exhibits finer and more uniform grains. The alloy with TiO_2_ film deposited by sol-gel exhibits better corrosion resistant. Nezamdoust et al. (2018) demonstrated that the silica coating prepared on the surface of AM60B Mg alloy by sol-gel method improved the surface roughness, corrosion resistance, and the hydrophobicity. Hu et al. (2011) used sol-gel method to prepare a nano TiO_2_ coating on AZ31 alloy, and reported that the degradation rate was lower when the size of nano-spherical TiO_2_ particles was smaller. Omar et al. (2020) synthesized 58S and 68S bioactive silica glasses by the sol-gel method, and dip-coated them on the AZ91D alloy. Results showed that the coatings improved corrosion resistance in Hank’s solution, and the cells were well adhered, spread, and elongated on the coated materials.

##### 3.2.1.5 Ion implantation

**FIGURE 7:**
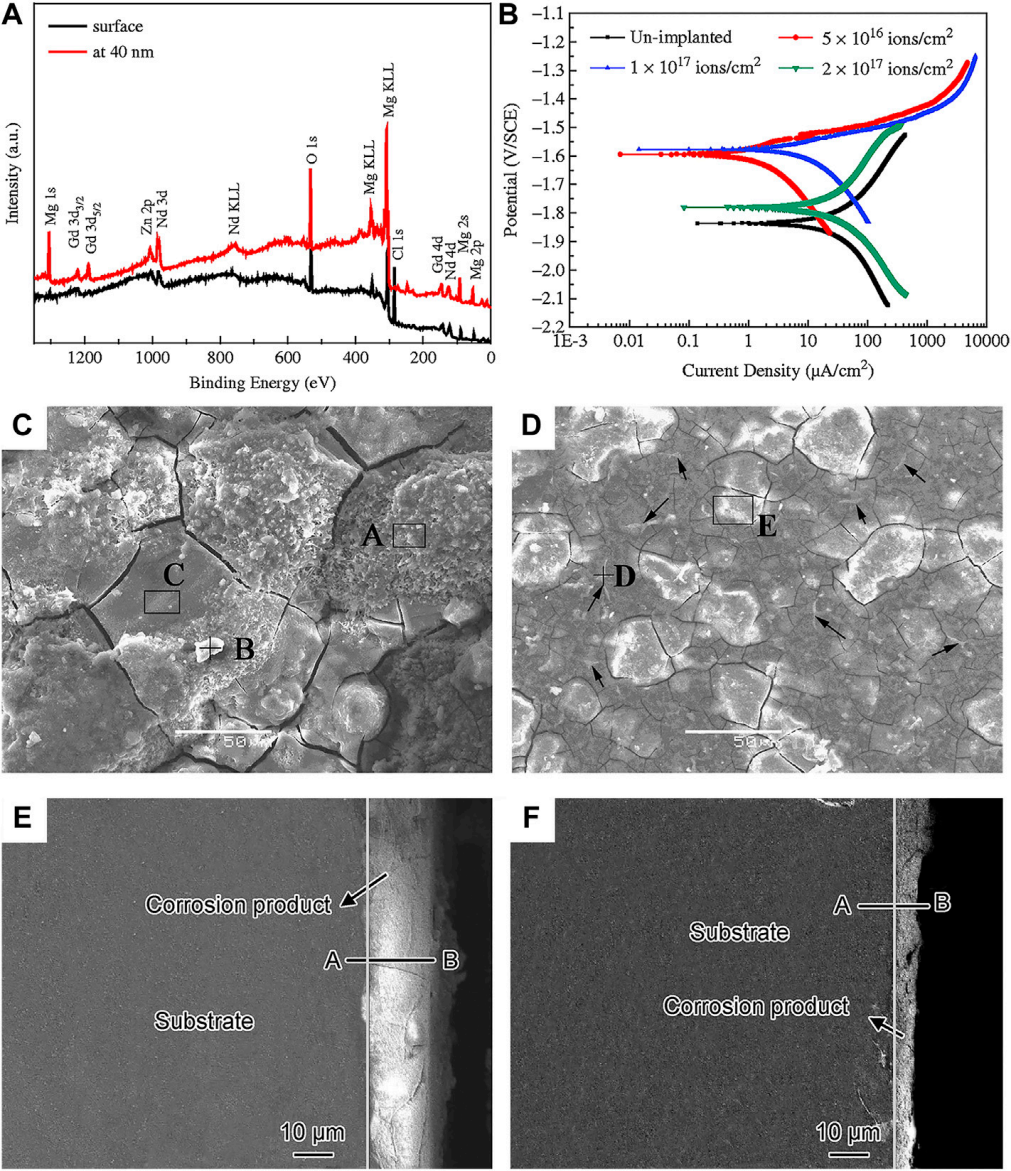
**(A)** XPS spectra of implanted alloy at different depths; **(B)** polarisation curves of Mg-Gd-Zn-Zr alloy with various doses in SBF solution; surface and cross-section morphology after corrosion test **(C,E)** unimplanted and **(D,F)** Nd implanted alloy. Reprinted with permission from reference (Wang et al., 2015b), Elsevier.

Ion implantation is a surface modification technique in which target elements are formed into an ion beam in a vacuum, and then sputtered onto the modified material, and finally a layer with specific composition and structure is formed on the substrate surface (Jamesh et al., 2014). Implantation of appropriated ions into Mg substrate can reduce its corrosion rate and improve mechanical performances and biocompatibility. Recently, the main researches include metal ion such as iron (Fe), cerium (Ce), zinc (Zn), zirconium (Zr), strontium (Sr), as well as the non-metallic ions such as carbon (C), oxygen (O), Nitrogen (N) (Liu et al., 2017; Somasundaram et al., 2018; Zhu et al., 2018). Jia et al. (2018) observed that after implanting Sr ions into Mg alloys, the elastic modulus and hardness are improved, and meanwhile the corrosion potential is increased. Zhu et al. (2018) implanted N ions on the surface of AZ31 alloy, followed by magnetron sputtering to generate double-layer amorphous hydrogenated diamond-like carbon (DLC:H)/SiNx, which effectively improves the long-term corrosion resistance of the substrate. Wang et al. (2015b) implanted Nd ions on the Mg-Gd-Zn-Zr alloy, and found that a mixed layer consist of Nd_2_O_3_, Gd_2_O_3_, and metal Nd was produced on the surface (Figure 7A). The Nd_2_O_3_ and Gd_2_O_3_ are relatively stable in the aqueous solution, preventing the inward transport of Cl^−^, which can increase the corrosion resistance of the alloy under the appropriate condition (Figure 7B). Figures 7C–F show the surface and cross-sectional morphologies of the corrosion product layer on the alloy before and after ion implantation. It is worth noting that the thickness of the corrosion product layer becomes smaller after ion implantation, and the alloy is uniformly etched before and after implantation. Liu et al. (2017) modified Mg alloys by metal vapor vacuum arc plasma deposition after implanting Zn ions on the surface of Mg-1Ca alloys. A relatively uniform ZnO coating was produced on the Mg alloy surface, which promoted the proliferation and adhesion of MC3T3-E1 cells and significantly increased the corrosion potential.

As one of the most important methods for surface modification of Mg alloys, ion implantation is convenience, controllability, and flexibility. Currently, single ion implantation is limited in improving the performance of materials. The implantation of ions with various functions can not only effectively improve the physical and chemical properties or biological activity of alloys, but also improve the antibacterial ability. With the development of composite ion implantation technology, it will be a trend to simultaneously implant multiple ions to obtain multifunctional Mg materials.

#### 3.2.2 Surface microstructural modification

Surface microstructural modification technique induces the deformation of the metal surface through mechanical processing, so that the material surface obtains a different microstructure and performance from the matrix material. Mechanical processing improves the mechanical performances and corrosion resistance of Mg alloys by refining grains, changing the distribution of second phases or intermetallic compounds, and enhancing surface hardness (Yin et al., 2020). The process usually does not involve chemical reactions. This part mainly introduces the research status of surface mechanical attrition, shot peening, laser surface modification, and friction stir processing (FSP) in improving the performances of Mg alloys for biomedical applications.

##### 3.2.2.1 Surface mechanical attrition

**FIGURE 8:**
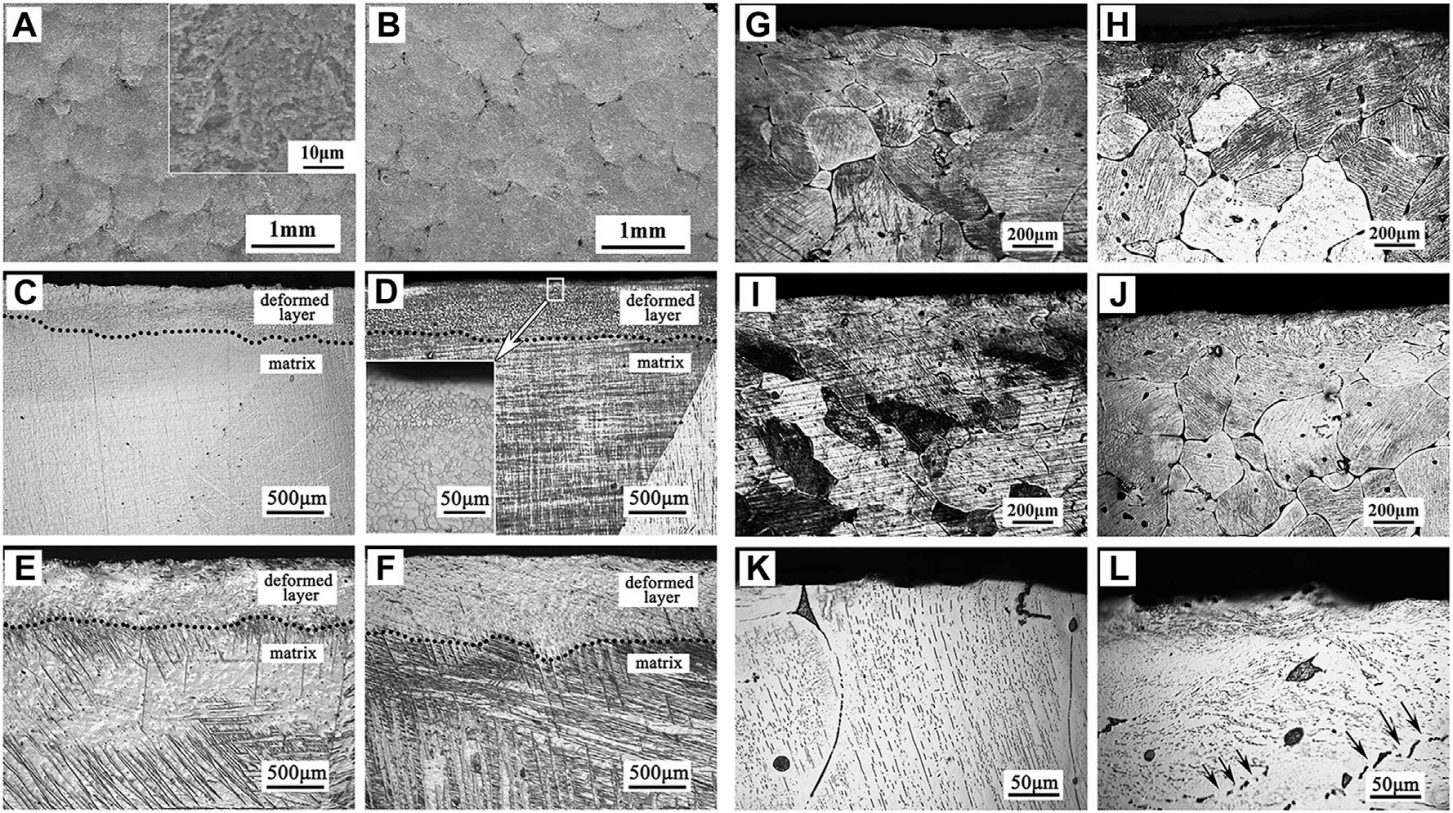
Surface morphologies of **(A)** SMATed pure Mg, and **(B)** SMATed Mg-1Ca alloy; cross-section morphologies of **(C–F)** SMATed pure Mg for different time, **(G–J)** SMATed Mg-1Ca alloy for different time, and high magnification images of **(K)** untreated and **(L)** SMATed Mg-1Ca alloy. Reprinted with permission from reference (Chen et al., 2019a), Elsevier.

Surface mechanical grinding treatment (SMAT) is a promising surface nanocrystallization technique, which can refine grains to nanoscale and form gradient nanostructures without changing the composition of materials. It has a significant effect on the improvement of the corrosion resistance of Mg alloys. After SMAT, the microstructure of Mg alloys is fine and uniform, the surface is relatively smoother, and the corrosion rate is significantly reduced (Xia et al., 2016). The surface and cross-section morphologies of Mg and Mg alloy after SMAT as indicated in Figure 8. For instance, Laleh and Kargar (2011) performed SMAT process on AZ91D Mg alloy and found that the corrosion rate was significantly reduced. However, studies have been shown that the degradation resistance of Mg after SMAT decreases due to the increased crystal defect density after grain refinement and surface contamination caused by the attrition balls (Li et al., 2014; Skowron et al., 2020). Similarly, Chen et al. (2019a) reported that the H_2_ release, weight loss, and the corrosion rate of the alloy after SMAT were twice as high as those of the untreated alloy due to the increased surface roughness. In summary, it can be considered that SMAT is less effective for improving the performances of biomedical Mg alloys. In the future, composite technology should be developed towards combining SMAT with other effective surface coating techniques.

##### 3.2.2.2 Shot peening

**FIGURE 9:**
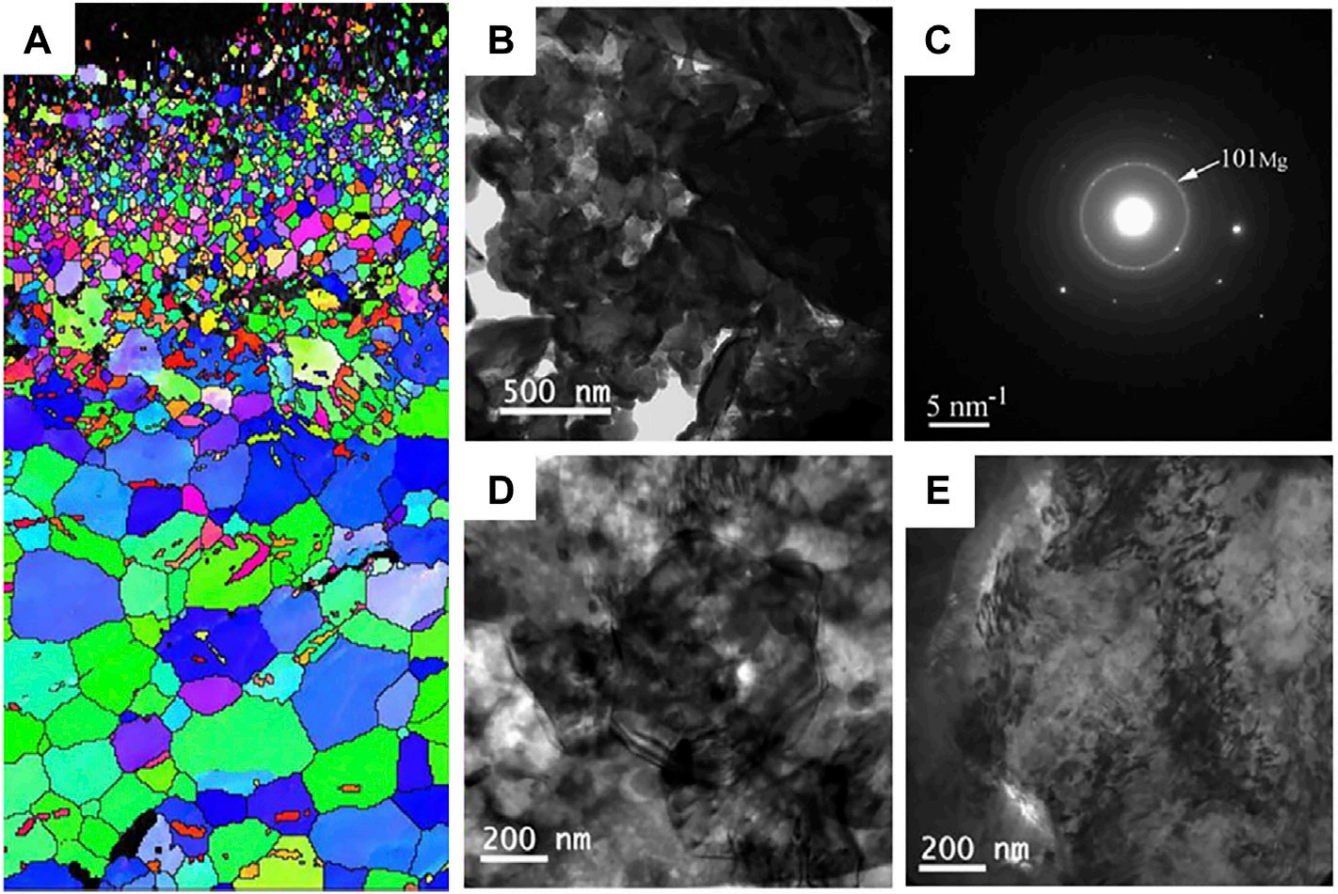
**(A)** EBSD map of the cross section of AZ31 samples after shot peening; TEM images **(B)** bright field image and **(C)** the corresponding SAED pattern of the shot peening sample just under the topmost layer, bright field images at the depth of **(D)** 30 μm and **(E)** 150 μm. Reprinted with permission from reference (Bagherifard et al., 2018; Bagherifard et al., 2019), Elsevier.

Shot peening is also a common surface modification technique that introduce compressive residual stress to the Mg surface through a similar principle to SMAT technique. The plastically deformed zone formed by the shot peening process has an extended and refined grain structure (Kovacı et al., 2019). The representative microstructure of Mg alloys after shot peening is show in Figure 9. Mhaede et al. (2014) found that shot peening is a good way to improve microhardness and degradation resistance by refining grains and increasing the density of coating. Similarly, Yao et al. (2021) performed shot peening on AZ91 alloys after zinc coating and demonstrated that shot peening increased the densification of Zn coating, increasing the microhardness, and corrosion resistance. The H_2_ release and weight loss were reduced significantly for the shot-peened Zn-coated samples, compared to the bare sample. In addition, Bagherifard et al. (2018) investigated the effects of sever shot peening on the properties of AZ31 Mg alloy. They observed higher i_corr_ values, the cell viability was no obvious improvement for different shot-peened samples, which could be attributed to the rough surface layer after shot peening. Similarly, Peral et al. (2020) also demonstrated that higher surface roughness during shot peening promoted the rapid degradation. Therefore, similar to SMAT, shot peening is limited for improving the biological function of Mg alloys. In the future, we should focus on the development of composite techniques combining shot peening and coating preparation processes.

##### 3.2.2.3 Laser surface modification

**FIGURE 10:**
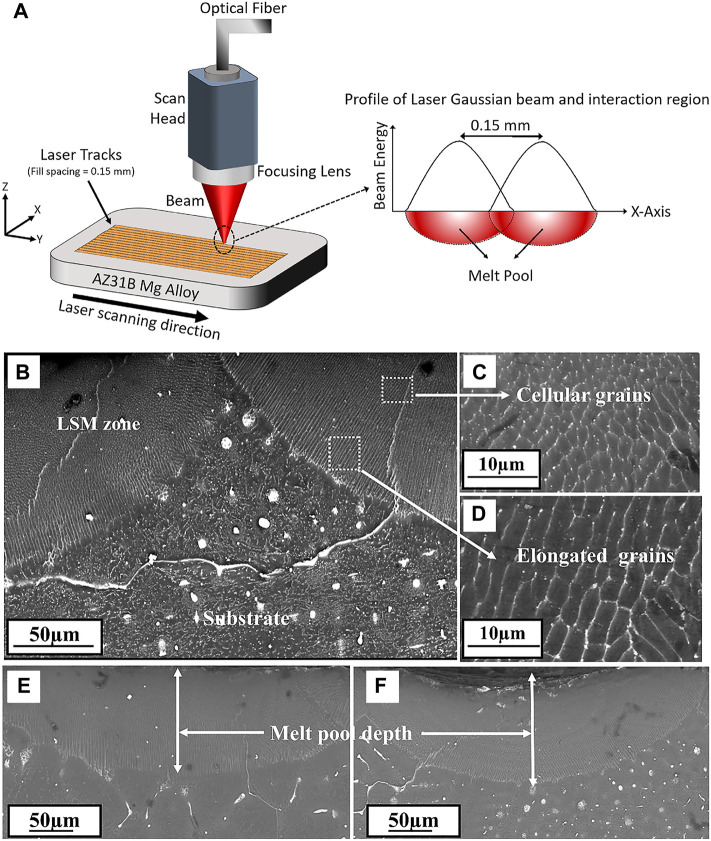
**(A)** Schematic of laser surface modification process; SEM images of cross section microstructure **(B)** laser melted Mg-Zn-Dy alloy, **(C)** and **(D)** enlarged views, **(E)** and **(F)** meltpool depth of the alloy processed at different conditions respectively. Reprinted with permission from reference (Rakesh et al., 2019), Elsevier.

Laser surface modification technology is an effective method to modify the material surface through melting by high intensity laser beam due to its high efficiency, no pollution, and low material consumption (Liu et al., 2020b; Hafeez et al., 2021). The schematic of laser surface modification is illustrated in Figure 10A. After laser surface modification, the microstructure of the alloy surface is changed significantly, such as the formation of fine dendritic grain layer without obvious porosity (Figures 10B–E). Nowadays, laser surface modification, mainly including the laser surface melting (Mistry and Vadali, 2022), laser cladding (Gao et al., 2022), and laser surface alloying (Yang et al., 2022), have been extensively applied in surface engineering.

In particular, Among these techniques, laser surface melting has attracted extensive attention to enhance the mechanical performances and corrosion resistance of Mg alloys due to the absence of additional alloying elements and a natural metallurgical bonding interface (Afzal et al., 2015). Numerous studies have been conducted on the microstructure and mechanical performances of Mg alloys after LSM treatment. For instance, Wu et al. (2021) used LSM for surface modification of AZ31B alloy, and found that the grain growth along the Z direction and form a cellular/dendritic microstructure, with the Mg_17_Al_12_ phase uniformly distributed along the grain boundaries. Liu et al. (2015a) also found that the microstructure of AM60B Mg alloy was composed of refined α-Mg grains and uniformly distributed secondary phases after laser surface melting. Guan et al. (2010) observed that the β-Mg_17_Al_12_ phases refined and Al concentration increased in AZ91 Mg alloy after laser melting, causing a decrease of corrosion rate by 70%.

**FIGURE 11:**
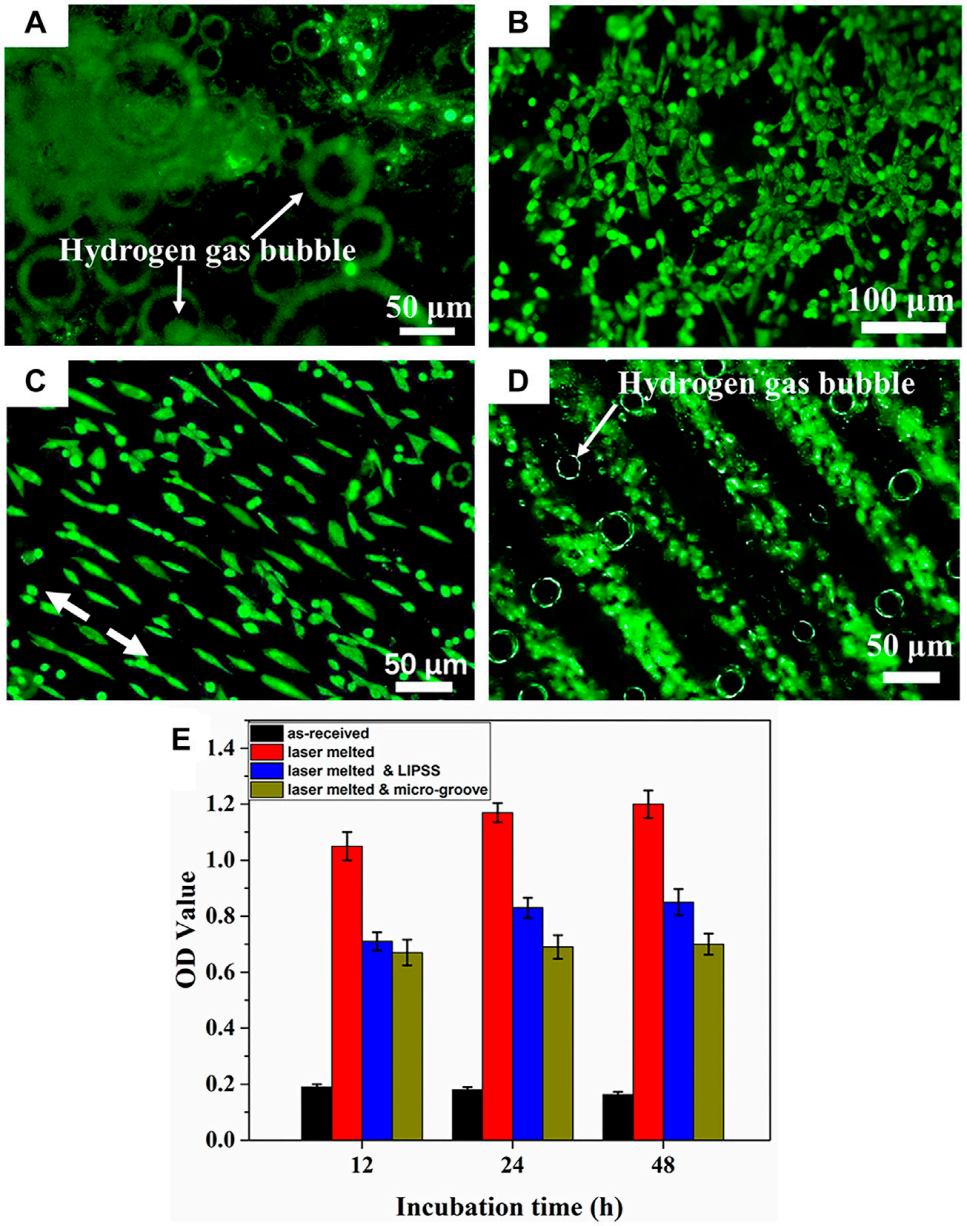
Fluorescence images of the MC3T3-E1 cell on **(A)** as-received, **(B)** laser melted, **(C)** laser melted and LIPSS, **(D)** laser melted and micro-groove surface and **(E)** cell proliferation after cultured for 48 h. Reprinted with permission from reference (Zhang et al., 2019), Elsevier.

In addition, the effect of surface texture formed during the LSM process on the degradation behavior of Mg alloys has been reported by Zhang et al. (2019). The LSM-treated Mg alloys not only improved mechanical performances and the degradation resistance, but also promoted cells adhesion and proliferation along the direction of LSM-induced nanotexture (Figure 11). Manne et al. (2018) investigated the effect of different laser powers and scanning speeds of LSM on Mg-2.2Zn alloy, and found that the most refined morphology was obtained at a power of 125 W and a scanning speed of 30 mm/s. The corrosion rate of Mg-2.2Zn alloy in HBSS was reduced by more than 40%, and the biomineralization was improved due to the enhanced surface energy for the LSM-treated substrate. In summary, laser surface modification can improve the cytocompatibility and corrosion resistance of Mg alloys by improving the surface microstructure of the substrate, efforts should be devoted to optimizing the process parameters and carrying out more in-depth research in the future.

##### 3.2.2.4 Friction stir processing

A microstructural modification method called friction stir processing (FSP), with a principle similar to friction stir welding (FSW), which is developed by Mishra (Mishra and Ma, 2005), is generally used for mechanical property enhancement and surface composite fabrication of light alloy (Cao et al., 2018; Gu et al., 2019; Wang et al., 2020b). FSP is an effective technique that can produce fine grains and uniform microstructures and improve mechanical performances in base materials (Wang et al., 2015a; Wang et al., 2017). Darras et al. (2007) obtained fine grains and homogenous microstructure by a single-pass FSP. Similarly, Cavaliere and De Marco (2007) observed that the AZ91 alloy exhibited superplasticity with an elongation of 1,050% at 300°C and 10^–4^ s^−1^ due to the grain refinement after FSP. In addition, FSP is also beneficial to the corrosion resistance of Mg alloys, which has been applied to optimize surface microstructure or to prepare a composite layer on the substrate surface in biomedical field (Liu et al., 2018b; Liu et al., 2019a). For instance, Zhu et al. (2013) found that the Mg-Zn-Y-Nd alloy after FSP exhibited uniform equiaxed fine grains (∼5 μm), and the intermetallic compounds were refined to nanoscale due to the dynamic recrystallization. Similarly, Liu et al. (2018b) obtained the fine grains and intermetallic compounds in AZ91 Mg alloy by FSP (Figure 11). Besides, they investigated the corrosion resistance of AZ91 alloy before and after FSP, and observed that a dense and continuous *β* phase layer was formed on the surface of the FSPed sample due to the segregation of fine *β* phase, which effectually improved the stability and passivity of corrosion product film. Argade et al. (2012) observed that the pitting corrosion potential of Mg-Y-RE alloy after FSP is higher than that of base metal because of the grain refinement and uniform second phase distribution during FSP.

Recently, a large number of researches have been carried out on the fabrication of surface metal composites by FSP (Ding et al., 2016; Saikrishna et al., 2018; Yang et al., 2018; Wang et al., 2021). Three ways of adding reinforcing particles to the alloy surface have been reported (Zhang et al., 2017a; Ran et al., 2018; Wang et al., 2020c; Wang et al., 2022). One is to fill the processed grooves or holes with reinforcing particles, the other is to fill the reinforcing particles between two plates to form a sandwich-like structure, and the third is to pre-assemble the reinforcing particles in a stirring tool with a hollow structure. Generally, surface composites exhibit the combination of grain refinement by FSP and reinforcement particle when the reinforcing phase is incorporated into a substrate (Qin et al., 2018; Qiao et al., 2021). For instance, Jiang et al. (2013) fabricated nano-SiO_2_/AZ31 Mg composites with FSP and found the addition of nano-SiO_2_ could refine the grain to nanoscale and increase the composite hardness.

**FIGURE 12:**
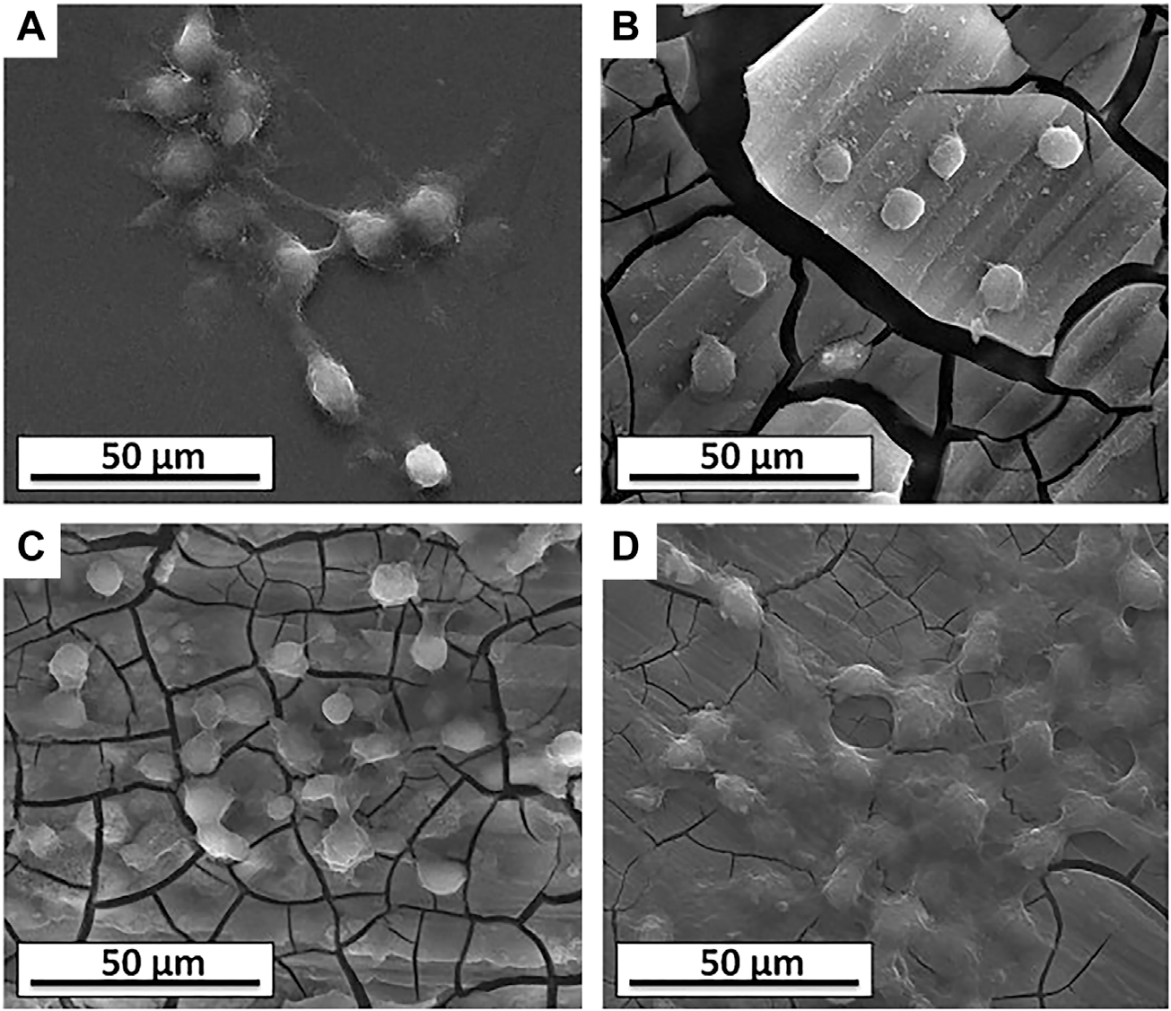
SEM morphologies of the L6 cells on the surface of the samples: **(A)** control, **(B)** Mg, **(C)** FSP-Mg, and **(D)** FSP-Mg-nHA. Reprinted with permission from reference (Sunil et al., 2014a), Elsevier.

Nowadays, Sunil et al. (2014a) and Sunil et al. (2014b) used FSP to embed the nano-hydroxyapatite (nHA) powder (∼32 nm) on the Mg surface and successfully prepared a fine-grained Mg-nHA composites for degradable bone implants. After FSP, the grains are obvious refined and the nHA distribution is uniform at the surface of pure Mg. Compared with unprocessed Mg, the Mg-nHA composite exhibits superior bioactivity from *in vitro* bioactivity tests because the presence of apatite formed from nHA particles on the composite surface enhances the biomineralization and reduces the corrosion rate. Cell culture studies indicate that the better cell adhesion and proliferation on the FSP-Mg-nHA composites compared with unprocessed Mg and FSP-Mg (Figure 12). Similarly, Hanas et al. (2018) prepared HA-enhanced AZ31 composites by FSP to obtain better biological activity. Qin et al. (2018) promoted the uniform dispersion of hydroxyapatite to the ZK60 Mg alloy by two-pass FSP, and obtained a surface composite with enhanced corrosion resistance. Similarly, Qiao et al. (2022) used multi-pass FSP to prepare the ZrO_2_ particles reinforced AZ31 Mg composites, and found that with the increase of the pass, the distribution of strengthening particles was more uniform, and the mechanical performances and corrosion resistance were significantly improved.

As a solid-state microstructure modification technology, FSP plays a great role in improving the properties of Mg alloys. On the one hand, the grains are refined and surface defects are eliminated due to the sever plastic deformation during FSP. On the other hand, it can be combined with other technologies or reinforcement materials to simultaneously improve the mechanical and biological properties of materials. In summary, it is a promising research direction for developing FSP technology to modify the microstructure and prepare Mg-based composites for biomedical applications.

## 4 Conclusion

Mg alloys are considered to be a promising biodegradable implant material due to their biodegradability, good biocompatibility, and biomechanical compatibility, which is should be further investigated to develop the Mg and Mg alloys for biomedical applications. This review mainly summaries the degradation mechanism of Mg alloys under the action of various condition and stress, and the commonly emphasized surface modification methods, such as chemical conversion, micro-arc oxidation, sol-gel, ion implantation, surface mechanical attrition, shot peening, laser surface modification, and FSP. For biomedical Mg alloys, the rapid degradation rate, and insufficient mechanical performances limit their clinical application as load-bearing parts. Therefore, it is necessary to clarify the degradation mechanism of Mg alloys under the action of complex condition and stress. Secondly, it is need to realize the controllability of the degradation rate of Mg alloys on the basis of ensuring biocompatibility and safety. Ultimately, it should be combined with surface modification technology to improve the mechanical performances and corrosion resistance in the future.
